
JOURNAL INFORMATION
==============================
NLM Title Abbreviation: Innov Surg Sci
Journal ID: Innov Surg Sci
NLM Journal ID: 101708165
Journal ID: iss

Innovative Surgical Sciences

EISSN: 2364-7485
Publisher: De Gruyter

ARTICLE INFORMATION
==============================
PMCID: PMC10216883
DOI: 10.1515/iss-2023-9007
Article ID: iss-2023-9007
Article version: 1
Subjects: Article

Abstract DGU

Publication date: 2023 Mar
Electronic publication date: 2023 Mar 13
Volume: 8
Issue: Suppl1
First page: 137
Last page: 147
Copyright: © the Author(s), published by De Gruyter
Copyright year: 2023
Copyright holder: the Author(s), published by De Gruyter
License: This work is licensed under the Creative Commons Attribution 4.0 International License.
License URL: https://creativecommons.org/licenses/by/4.0/

Page count: 11

==============================

### Incidence and treatment of proximal humeral fractures in the elderly during the COVID-19 pandemic in Germany – A health claims data analysis

ID: 370

**Kategorie: DGU - Alterstraumatologie**

Jeanette Köppe[1], Josef Stolberg-Stolberg[2], Karen Fischhuber[1], Janette Iking[2], Ursula Marschall[3], Andreas Faldum[1], Michael Raschke[2], J. Christoph Katthagen[2]

[1]*Westfälische Wilhelms-Universität Münster, Institut für Biometrie und Klinische Forschung, Münster, Deutschland*, [2]*Universitätsklinikum Münster, Klinik für Unfall-, Hand- und Wiederherstellungschirurgie, Münster, Deutschland*, [3]*BARMER Institut für Gesundheits-systemforschung, Wuppertal, Deutschland*

**Background:**

The COVID-19 pandemic particularly affected elderly patients with higher risks for a severe course of infection and aggravation of secondary diseases. The aim of this study was to describe the incidence of proximal humeral fractures (PHFs) during the lockdowns of the pandemic in Germany and to assess complications and mortality of patients with simultaneous COVID-19 infections.

**Materials and methods:**

Health claims data of the German BARMER Health Insurance from 01/2005 – 11/2021 were retrospectively analysed. In the beginning of 2023, however, a data update until 11/2022 will be available. All inpatient cases of elderly patients (age ≥ 65 years) with a primary or secondary diagnosis of PHF (ICD: S42.2) were included into the study and the incidence of PHF per 100,000 was determined. Differences between cases of PHF with and without existing SARS-CoV-2 infection (defined using ICD U07.1, U07.2) for various in-hospital outcomes (length of hospital stay [LOS], charges, thromboembolic events, major adverse events [MAE], and death from any cause) were analysed descriptively and risk adjusted using multivariable linear and logistic regression models.

**Results:**

In total, n=130,810 (84.5% female sex, median age 79 years) cases with PHF as primary or secondary diagnosis were included to the study, with 51.6% of them receiving surgical fracture fixation (22.5% locked plate fixation and 7.6% reverse total shoulder arthroplasty). From 2010 to 2019, the incidence of PHF (age ≥ 65 years) was comparable with a mean value of 353.8/100,000 person years, while the incidence for female patients was much higher (female 465.4/100,000 vs. male 153.4/100,000). In 2020, however, the incidence of PHF decreased to 332.3/100,000 person years. In more detail, during the first lock-down in 04/2020, the 1-month incidence of PHF per 100,000 decreased to 25.3, compared to the mean incidence in 04/2017–2019 of 29.3/100,000 person months. Similar trends were observed during the winter of 2020/21: the incidence of PHF observed was 29.2, 28.6, 28.4 and 30.8 per 100,000 person months for 10/2020, 11/2020, 12/2020 and 01/2021, respectively (mean incidence per month of 10/11/12/01 2017 – 2019: 32.2, 32.5, 32.9 and 36.3/100,000 person months).

Open Access. © 2023 The Author(s), published by De Gruyter. This work is licensed under the Creative Commons Attribution 4.0 International License.

However, no relevant trends for the ratio of cases with surgical treatment of PHF were found. During the study, n=517 cases with SARS-CoV-2 infection were detected. In cases with COVID-19 infection, patients were older (median (Q1, Q3) age: 82 (77, 87) years vs. 80 (73, 85) years), showed a worse comorbidity profile – e.g. suffered more often from diabetes (23.4% vs. 18.2%), chronic kidney disease (23.0% vs. 16.1%), or chronic heart failure (22.4% vs. 10.4%) – and received less often surgical treatment (47.8% vs. 54.5%). Furthermore, COVID-19 infection was associated with higher mean costs (risk-adjusted mean differences (95%CI) 907.45 EUR (192.2 – 1622.7 EUR); p=0.013) and longer LOS (risk-adjusted mean differences (95%CI) 6.37 days (5.6 – 7.2 days); p<0.001). Furthermore, in PHF-cases with COVID-19 infection, higher rates of death (12.3% vs. 2.3%) and MAEs (19.7% vs. 7.3%, both p<0.001) were observed, which persisted even after risk-adjustment: COVID-19 infection was associated with higher risk for death (odd ratio [OR] 4.06, 95%CI 2.90 – 5.70, p<0.001) and MAEs (OR 1.90, 95%CI 1.47 – 2.45, p<0.001) during hospital stay. However, after adjustment, no excess of mortality was detected for the pandemic years alone (all p>0.05).

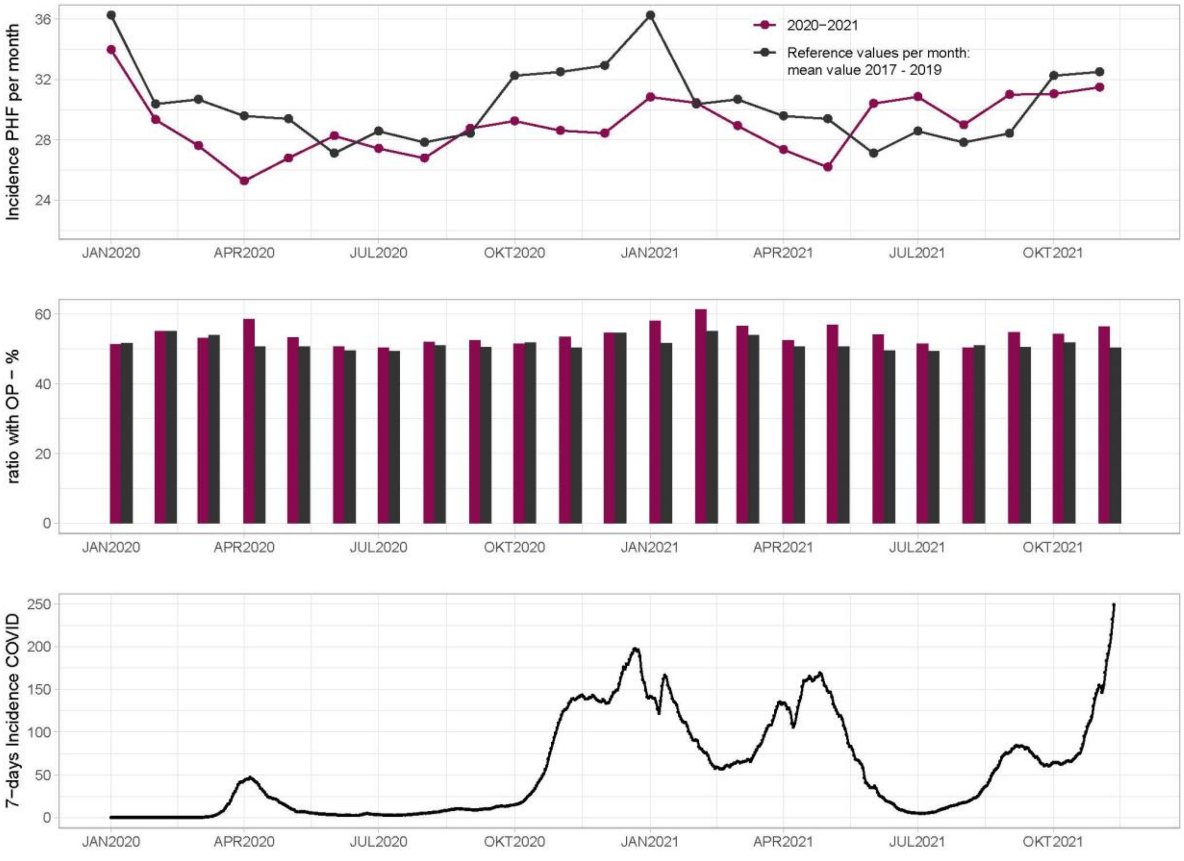

Figure 1: Time trends of incidence of PHF per 100,000/ person months and ratio of surgical treatment over COVID-19 pandemic. 7-days incidence for COVID-19 was given by the data of the Robert-Koch institute (www.rki.de/inzidenzen, as of 11.09.2022).

**Conclusion:**

During the lockdowns of the initial phase of the COVID-19 pandemic, there was a significant decrease in the incidence of PHF. Less mobility of the elderly, fewer hospitalizations despite injury and changes in care structures might be some causes for this observation. Our data underline that patients at risk are particularly the elderly with secondary disease. Patients with a PHFs and an active COVID-19 infection bear a great risk for increased complications and mortality during hospital stay.

**Outcome after proximal humeral fracture in the elderly – non-surgical compared to surgical treatment**
ID: 382

**Kategorie: DGU - Alterstraumatologie**

Jeanette Köppe[1], Karen Fischhuber[1], Josef Stolberg-Stolberg[2], Janette Iking[2], Ursula Marschall[3], Andreas Faldum[1], Michael Raschke[2], J. Christoph Katthagen[2]

[1]*Westfälische Wilhelms-Universität Münster, Insitut für Biometrie und Klinische Forschung, Münster, Deutschland*, [2]*Universitätsklinikum Münster, Klinik für Unfall-, Hand- und Wiederherstellungschirurgie, Münster, Deutschland*, [3]*BARMER Institut für Gesundheits-systemforschung, Wuppertal, Deutschland*

**Background:**
The proximal humeral fracture (PHF) of the elderly is the third most common osteoporotic fracture and a major public health concern due to a rising incidence along with demographic changes. While approximately two thirds of all PHFs are treated conservatively, surgical treatments including locked plate fixation (LPF) and reverse total shoulder arthroplasty (RTSA) are recommended in cases of displacement or multiple-part fractures. However, there is no clear evidence for the benefit of surgical over conservative treatment of PHFs. Hence, the aim of this study was to evaluate and compare treatment outcomes in terms of complications and mortality of conservative treatment, LPF, and RTSA in elderly patients with PHFs.

**Materials and methods:**
Health claims data of the German BARMER Health Insurance from 01/2005 – 12/2021 were retrospectively analysed. All elderly patients (age ≥ 65 years) with a coded diagnosis of PHF (ICD: S42.2) from 01/2011 – 12/2020 were included. Patients were divided into four treatment groups according to the treatment they received within the first 21 days after diagnosis: (1) non-surgical treatment, (2) simple-fracture locked plate fixation (sLPF), (3) multi-fragmented locked plate fixation (LPF), and (4) RTSA. With a median follow-up period of 66.6 months, patients were observed from the time of the first diagnosis of PHF to the end of follow-up (defined by death from any cause, end of study (31/12/2021), or exit from the database). The individual entry time to the study, i.e. 21 days for patients with non-surgical treatment and time from PHF diagnosis to surgery, was considered in all analyses. Overall survival (OS), major adverse events (MAEs; defined as acute myocardial infarction, stroke, resuscitation, cardiac arrest, acute renal or liver failure, acute respiratory distress syndrome, sepsis, or death from any cause), and surgical events (SEs) were defined as primary outcomes. For OS and MAE, event rates after one year were determined using Kaplan-Meier estimates and differences between the treatment groups were tested via two-sided Log-rank test. For SEs, death was considered as a competing risk event and the cumulative incidence function was determined using Aalen-Johansen estimates. All presented results are unadjusted without multivariable risk-adjustment for patient's comorbidity profile.

**Results:**

In total, n=75,498 (median age 78 years, 83.8% female sex) patients were included into the study, with 25.6% of the patients receiving a surgical treatment of PHF during the first 21 days after diagnosis. During the study period, the proportion of patients treated with LPF decreased, while treatments with RTSA increased continuously (2011: non-surgical treatment 72.6%, sLPF 3.5%, LPF 21.7%, and RTSA 2.2% vs. 2020: non-surgical treatment 72.5%, sLPF 2.8%, LPF 12.7%, and RTSA 11.9%). Patients with conservative treatment had less often female sex (82.9% vs. 86.3%, 83.9%, and 87.5% for sLPF, LPF and RTSA, respectively) and were more often co-diagnosed with dementia (12.9% vs. 11.0%, 8.6%, and 9.4% for sLPF, LPF and RTSA, respectively). The proportion of patients with osteoporosis was highest in patients treated with RTSA (non-surgical treatment 33.5%, sLPF 30.6%, LPF 31.9%, and RTSA 35.2%), with 11.5% of the patients receiving anti-osteoporotic therapy before PHF (non-surgical treatment 11.5%, sLPF 11.5%, LPF 11.0%, and RTSA 12.1%).
One year after PHF, 10.6% (95%CI 10.4 – 10.9%) of the patients have died, while lowest OS were observed in patients treated with sLPF and RTSA (1-year rate of death [95%CI]: non-surgical treatment 9.7% [9.5 – 10.0%], sLPF 11.1% [9.7 – 12.5%], LPF 7.8% [7.3 – 8.3%], and RTSA 10.1% [9.3 – 11.0%]; p<0.001). During the first year after PHF, 11.7% (95%CI 11.4 – 12.0%) of the patients suffered from a MAE, again with the highest occurrence in patients treated with sLPF and RTSA (1-year rate of MAE [95%CI]: non-surgical treatment 10.7% [10.4 – 10.9%], sLPF 12.3% [10.8 – 13.7%], LPF 8.8% [8.3 – 9.3%], and RTSA 11.5% [10.6 – 12.4%], p<0.001).
Moreover, one year after PHF, 17.6% (95%CI 17.2 – 17.9%) of the patients that initially were treated conservatively had to undergo surgery after all, where LPF (41.5%) and other open reduction and internal fixation procedures (29.4%) were the most frequent treatment options after failed conservative treatment (RTSA 21.8%; sLPF 7.3%).

**Conclusion:**

Consistent with literature, the majority of patients were treated conservatively. Interestingly, patients after sLPF showed the lowest OS and highest complication rate indicating that the decision for surgical treatment has to be made very carefully even in cases of simple fractures. High rate of osteoporosis in the RTSA group indicates that complex fracture patterns make a total joint replacement inevitable. However, this might be prevented with a more consequent anti-osteoporotic therapy beforehand.

**The surgical management of highly unstable fragility fractures of the sacrum with spinopelvic dissociation: A case series and proposal of a surgical treatment algorithm**

ID: 850

**Kategorie: DGU - Alterstraumatologie**

Andrea Gross[1], Christoph Meier[2]

[1]*Kantonsspital Winterthur, Kantonsspital Winterthur, Winterthur, Schweiz*, [2]*Kantonsspital Winterthur, Orthopädie und Traumatologie, Winterthur, Schweiz*

**Background:**

U- and H-shaped fragility fractures of the sacrum (FFP IVb)) are unstable fractures. Non-operative treatment may be associated with impaired walking abilities, chronic pain and the potential loss of independency. However, different treatment options are still controversially debated. The aim of surgical treatment includes sufficient fracture stability for immediate full weight bearing and good pain control postoperatively. A new surgical treatment algorithm was developed. This algorithm was evaluated in a cohort of geriatric patients with FFP type IVb regarding surgical complications and patient-related outcome.

**Materials and methods:**

Retrospective case series. Patients ≥65 years, admitted with FFP IVb between 01/2017 and 12/2020 were included. Pelvic CT was performed and the surgical technique was chosen according to the proposed surgical treatment algorithm. For this algorithm, the technique of fracture stabilisation was adapted to sacral anatomy and the specific fracture pattern to minimize the impact of surgery and postoperative complications without compromising a sufficient fracture stability. Pain levels, complications and surgical revisions were recorded. Level of independency, walking abilities and -aids were compared 3- and 12- months postoperatively to the pre-injury situation.

**Results:**

Twenty-three patients were included. Outcome parameters could be obtained in 20 patients (85%) after three months and in 18 patients (78%) patients one year postoperatively. All patients were treated according to the algorithm. Sixteen patients received two TI-TSS, whereas either lumbopelvic fixation (LPF) combined with a TI-TSS or bilateral ISS was performed four times. Three patients underwent bilateral ISS into S1 with one TI-TSS into S2. Three days postoperatively, median pain VAS was 2 (0-8) compared to 7 (4-10) before surgery. One loosened TI-TSS was removed six weeks postoperatively. Three-month mortality was 14% (n=3). At one-year follow-up, all patients regained their pre-injury level of walking abilities.

**Conclusion:**

Restoration of walking abilities, preservation of independency and efficient pain control can be achieved with surgical fixation of FFP type IVb fractures. With the proposed surgical algorithm, the fixation techniques are adapted to the fracture pattern to minimize the surgical burden.

**Prediction of postoperative mobility in patients with pertrochanteric femur fractures using the prognostic nutritional index**
ID: 863

**Kategorie: DGU - Alterstraumatologie**

Leon Faust[1], Maximilian Lerchenberger[1], Johannes Gleich[1], Alexander Keppler[1], Carl Neuerburg[1], Yunjie Zhang[1]

[1]*Muskuloskelettales Universitätszentrum München , LMU Klinikum, München, Deutschland*

**Background:**
Postoperative early mobilization is critical to prevent postoperative complications in elderly patients with hip fractures. Postoperative mobility varies vastly among orthogeriatric patients. To predict postoperative mobility, it is essential to take multiple and patient-specific factors into account. Malnutrition can be frequently found in geriatric patients and was shown to be a risk factor for postoperative complications. The prognostic nutritional index (PNI) has been widely used as an indicator to assess the nutritional and immunological condition of patients. This study aimed to investigate the predictive value of the PNI for early postoperative mobility in geriatric patients with pertrochanteric femur fractures. We hypothesized that patients with a lower PNI have impaired postoperative mobility.

**Materials and methods:**
Orthogeriatric patients (aged ≥ 65 years) who were diagnosed with pertrochanteric femur fracture, and received a TFN-Advance™ (TFNA, DePuy Synthes, Raynham, USA) implantation in our hospital between June 2020 and April 2022, were recruited. Several clinical parameters, together with the PNI, which was calculated using the postoperative routine blood analysis, were evaluated using univariable and multivariable logistic regression analyses. Mobility on the third postoperative day and mobility by the time of discharge were defined as endpoints. The receiver operating characteristic curve was used to estimate the optimal PNI cut-off value for mobility on the third postoperative day. Patients who were able to mobilize with a walker, a walking frame, on crutches, or freely were considered mobile. Patients who could only stand or were bedridden were defined as immobile.

**Results:**
In total 156 patients were evaluated. The stepwise logistic regression showed that PNI (OR: 1.14, 95% CI: 1.07-1.23, $p < 0.01$) was a predictor of mobility on the third postoperative day; PNI (OR: 1.18, 95% CI: 1.08-1.30, $p < 0.01$) and dementia (OR: 0.17, 95% CI: 0.07-0.40, $p < 0.001$) were predictors of mobility by discharge. PNI was weakly associated with age (r2 = 0.07, $p < 0.001$). The cut-off value of PNI for mobility on the third postoperative day was 38.1 (specificity=78.5%, sensitivity=63.6%).

**Conclusion:**

Our findings showed that PNI was an important patient-specific factor to predict early postoperative mobilization in patients with a pertrochanteric femur fracture treated with TFNA™. Further, it was found that dementia, together with PNI, significantly influenced the mobilization at the end of the hospital stay. The results show that malnutrition, assessed with PNI, might serve as a predictive factor for reduced postoperative mobility in elderly patients. Subsequently, studies should investigate the potential beneficial impact of perioperative nutrition supplements on postoperative mobility.

**LiBoD – Liquid Biopsy in Organ Damage**

ID: 392

**Kategorie: DGU - Polytraumaversorgung**

Bingduo Wang[1], Aliona Wöhler[2], Cindy C. Keller[1], Sebastian Schaaf[2], Robert Schwab[2], Christian P. Strassburg[1], [#]Veronika Lukacs-Kornek[3], [#]Arnulf G. Willms[2,3,4] and [#]Miroslaw T. Kornek[1,2]

*[1]Department of Internal Medicine I, University Hospital of the Rheinische Friedrich-Wilhelms-University, Bonn, Germany; [2]Department of General, Visceral and Thoracic Surgery, German Armed Forces Central Hospital, Koblenz, Germany; [3]Institute of Experimental Immunology, University Hospital of the Rheinische Friedrich-Wilhelms-University, 53127 Bonn, Germany; [4]Department of General, Visceral and Thoracic Surgery, German Armed Forces Hospital, Hamburg, Germany*

[#]shared last authorship

**Background:**

The aim of the study is non-invasively *via* a liquid biopsy to assess quality of trauma in polytrauma with organ rupture or laceration vs. polytrauma without internal organ damage. Extracellular vesicle (EVs) had been shown by us to be a suitable biomarker in other organ injuries as hepatic cirrhosis and liver cancer. Now, we aim to determine in polytrauma patients' serum concentrations of monocytes-derived small EVs, also named exosomes. To verify or to falsify our hypothesis that levels of monocyte-derived small EVs (sEVs) are elevated in polytrauma with organ injury. Here, we utilized NanoView Biosciences's ExoView Reader to capture sEVs for further analysis with commercially available antibodies and to measure their clinical performance.

**Materials and methods:**

CD14, CD44, CD44v6, CD61 surface antigens were confirmed on human THP-1 monocytes with and without differentiation to macrophages with PMA +/- LPS by FACS. sEVs from patients' serum were isolated by commercially available SEC columns (IZON70) followed up-concentration with 3kDa filters (Amicon). sEVs were captures with CD9, CD63 and CD81 on ExoView® chips according to manufacture instructions and individual sEVs subpopulations were analyzed subsequently for expression of CD14, CD44, CD44v6 and CD61. In brief, surface analysis of sEVs was performed with ExoView® R100 (NanoView Biosciences, Boston, USA) using their ExoView® Tetraspanin Kit (EV-TETRA-C). ExoView® chips were coated with CD81, CD63 and CD9 sEV capture antibodies before loading them with sEV samples. Antibodies were titrated prior use to ensure optimal performance. Data analysis was performed using ExoView® Analyzer 3.0. Only polytrauma patients with an injury severity score above 15 were included. Typically, serum samples were collected during 24hrs post traumatic event.

**Results:**

i) CD14, CD44, CD44v6 and CD61 were detectable on monocytes and predominantly on macrophages *in vitro*. ii) NTA and revealed a mean sEV size of 70nm in diameter. iii) CD14, CD44, CD44v6 and CD61 were differentially present on sEVs derived from prior to EV release differentiated THP-1 monocytes to macrophages with PMA and LPS. iv) sEV subpopulation of $CD9^{+}CD14^{+}$ sEVs were superior in identifying internal organ damage in polytrauma associated with a calculated cut-off of 3.7x10^5 $CD9^{+}CD14^{+}$ sEVs per mL serum (AUROC: 1.0, sensitivity: 100% and specificity: 100%). Followed by $CD9^{+}CD61^{+}$ sEVs (cut-off: 8.853x10^7, AUROC: 0.8867, sensitivity: 80%% and specificity: 80%). Other sEV subpopulations as $CD63^{+}CDxx^{+}$ or $CD81^{+}CDxx^{+}$ (CDxx=44, 44v6 and 61) were not conclusive. Correlation with ISS score was not conclusive. v) interestingly, limp amputees and arterial dissection led to highest CD14 and CD61 levels without internal organ damage. Hence, amputation must be regarded as an exclusion factor.

**Conclusion:**

Our preliminary data is in support of our initial hypothesis that the quality of trauma in polytrauma as organ rupture or laceration vs. polytrauma without internal organ damage might be determined *via* a liquid biopsy (LiBoD) during the first 24hrs post trauma event. This in particular from biggest interest for patients suffering from a blunt trauma that might pass out due to a blood volume decrease due to an unnoticed organ rupture.

*Studies were supported by German Armed Forces (Bundeswehr, project number 31K1-S-10 2023) the Deutsche Forschungsgemeinschaft (DFG, German Research Foundation) to M.T.K. (project number 410853455) and V.L.-K. (project number 411345524).*
